# Paving the way for systemic phenomenological psychiatry - the forgotten heritage of Wolfgang Blankenburg

**DOI:** 10.3389/fpsyt.2022.909488

**Published:** 2022-07-19

**Authors:** Samuel Thoma, Michael Konrad, Lisa C. Fellin, Laura Galbusera

**Affiliations:** ^1^Department of Psychiatry and Psychotherapy, Brandenburg Medical School, Immanuel Klinik Rüdersdorf, Rüdersdorf, Germany; ^2^Independent Researcher, Ravensburg, Germany; ^3^Department of Human and Social Sciences, University of Bergamo, Bergamo, Italy

**Keywords:** phenomenological psychopathology, systemic therapy, psychosis, phenomenological sociology, therapy of psychosis

## Abstract

Phenomenological psychopathology focuses on the first-person experience of mental disorders. Although it is in principle descriptive, it also entails an explanatory dimension: single psychological symptoms are conceived as genetically arising from a holistic structure of personal experience, i.e., the patient's being-in-the-world – and of its dynamic unfolding over time. Yet both classical and current phenomenological approaches tend to identify the essential disorder or “trouble générateur” (Minkowski) of mental illness within the individual, thereby neglecting the relevance of the social context not only for the emergence of symptoms but also for their treatment. The work of Wolfgang Blankenburg on schizophrenia represents a noteworthy approach to overcome this individualistic tendency. He introduced the concept of “loss of common sense” as the structural core of schizophrenic experience and being-in-the-world and he considered the social and most importantly familial context for the emergence of schizophrenic experience. By accounting not only for personal experience but also for interactional structures of families and social milieus in which experience is embedded, Blankenburg thereby offered ways to combine phenomenological and systemic explanations of mental disorders. Beside his most renowned work on “the loss of common sense,” in this paper we also present his family studies of young persons with schizophrenia, which have so far received little if no attention. We thus discuss the different ways in which Blankenburg expanded the phenomenological approach into a more systemic and social direction. We then link Blankenburg's work with current systemic explanatory models of schizophrenia and explore the clinical and scientific implications of this link. Finally, we call for further research on the synergy effects between the two.

## Introduction

Phenomenological psychopathology as a discipline aims at understanding so called mental disorders by focusing on and exploring first-person experience. As we will outline below in more detail, *understanding* mental disorders has been related to a merely descriptive endeavor, yet phenomenological psychopathology has also an explanatory goal. In the first part of this paper, we focus on what explanation means in phenomenological psychopathology and highlight how phenomenological explanatory modes might *per se* entail a systemic orientation. At the same time, we highlight one important limitation of most current phenomenological explanatory attempts within psychiatry, i.e., their individualism.

The central aim of this paper is to present the work of German psychiatrist Wolfgang Blankenburg on schizophrenia as a noteworthy approach to overcome this individualistic tendency. Blankenburg's research broadened the field of phenomenology into a theory of dialectics between self and world, with a strong emphasis on cultural and especially familial structures of interaction. Although Blankenburg's work is very renowned in the international scientific community, his later research on families of persons with schizophrenia has not received much attention. In this paper, we thus especially focus on Blankenburg's later writings.

In the last part of this paper, we look at Blankenburg's work through systemic lenses and emphasize several systemic aspects in his explanatory accounts of mental disorders, and of schizophrenia more specifically. We thus suggest that Blankenburg's work has expanded phenomenology – and phenomenological explanations – toward a systemic direction. We finally highlight the links between his research and the field of systemic therapy and point out opportunities for further exchange between current phenomenological and systemic thinking in psychiatry and mental health.

## Part 1. The explanatory dimension of phenomenological psychiatry

### The phenomenological method and the explanatory power of context

The connection between phenomenology and psychiatry has a long tradition that dates back to more than 100 years. Phenomenology attempts to reveal the intrinsic lawfulness of subjective experience independently of the idea of a general reality or external laws of nature. It thus claims that it is not possible to understand the domain of experience by making use of laws and principles that apply to the physical one (1).

Informed by Husserl's method, phenomenological psychiatry thus focuses on the domain of consciousness and subjectivity. It refuses the physicalist perspective of mainstream psychiatry that attempts to explain (the altered experience in) mental disorders to (supposedly objective) physiological dysfunctions at the neurobiological level of the brain. When it comes to mental disorders, the phenomenological approach thus does not focus on symptoms at a behavioral or physiological level but instead on the “conscious psychic event” (2), i.e., on patients' experience (3).

Jaspers (2), who was the first to introduce phenomenology into psychopathology, recognized the importance of an in-depth exploration of patients' first-person experience for the understanding of mental disorders. He introduced in psychopathology the seminal distinction between the epistemic modes of *explanation* and of *understanding*. On the one hand, the first refers to the establishment of causal relationships by means of repeated observations and thus mainly includes the physical realm of causality. More specifically, with the term *explanatory psychology* he characterizes approaches dealing with processes outside consciousness and the mechanisms through which they may determine conscious, psychic experience. Such “extraconscious mechanisms” are described as being essentially of somatic form (2). On the other hand, Jaspers speaks of *psychological understanding* as an emphatic and intuitive mode of knowledge, where experience is to be understood in its own terms. Here, Jaspers speaks of phenomenology as a descriptive psychology, i.e., as the *static understanding* of a person's subjective and conscious experience. On the whole, this approach results in the description of the present and lived condition, rather than on the extraconscious that determines these experiences (4). Contrary to static description of the conditions of experience, *genetic understanding* aims to trace the emergence of one psychic state from another (2). Genetic understanding consequently poses the question of the dynamic unfolding of one experience into the next and the meaningful unity in which they are contextualized.

Jaspers did not exclude explanation from the field of psychopathology and he rather argued for the need of methodological pluralism. Yet at the same time, by introducing for the first time the phenomenological method in psychiatry, he stressed the importance of focusing on experience *per se* and thereby on the domain of psychological understanding as the very foundation of psychopathology.

Many phenomenological authors thus have stressed that phenomenology – and phenomenological psychiatry - by only focusing on the experiential level has a merely descriptive and not explanatory function. However, based on the definition of phenomenological psychiatry as an *eidetic science*, starting from authors such as Binswanger and Minkowski (5), it is in fact possible to speak of an *explanatory dimension* of phenomenology both in terms of static and genetic understanding^1^. Interestingly, as we will now outline in more detail, explanation in phenomenological psychiatry is realized by the means of *contextualizing parts into a whole*.

*Eidos* or essence in phenomenology refers to a holistic structure or gestalt of different parts or elements of experience. This structure is neither independent of these elements or external to them nor can it be reduced to the mere sum of these elements–it indicates the way these elements relate to one-another as a comprehensive whole (5, 7, 8)^2^. Accordingly, eidos or essence here implies that what is merely factually experienced by the subject is anchored in more fundamental, more comprehensive structures of experience, i.e., someone's “being-in-the-world.”^3^ The Heideggerian concept of “being-in-the-world” (10) designates a comprehensive whole of experience composed of different elements such as world, mood (being-in) and understanding of our being. A single experience always thus occurs in further, implicitly experienced structures of this being-the-world. The aim of phenomenological psychiatry is to determine such concrete and immanent structures of experience in terms of a specific form of being-in-the-world of a patient. These structures determine single experiences and might thus play a causal role in the development of symptoms of a mental disorder (11).

Based on this concept of essence, whitin *static understanding* single experiences (such as perceiving an object or having certain feelings) are conceived as arising from the actual, present, and holistic structure of personal experience (i.e., the patient's being-in-the-world). The aim of static understanding is thus to grasp the current underlying common meaning and unity among subjective experiences, i.e., the eidos as structural and present state.

In contrast to the structural analysis of one's present state, *genetic understanding* strives at grasping the modification of the structure of experience over time, for instance by looking at a person's biography (12). Genetic understanding does not only concern–as in Jaspers–the emergence of one psychic phenomenon from another, but also and most importantly, how one (eidetic) form of being-in-the-world emerges from another and may thereby give rise to psychological symptoms. Sass and Parnas (11) recognize a sort of “autonomy of the phenomenological,” in that the very transformation of subjective experience can sustain and play a causal role in the development of experienced symptoms.

Far from being a merely descriptive endeavor, a phenomenological analysis can thus provide explanatory insights on the development of the disorder both in present state and over time. Moreover, by emphasizing that consciousness needs to be considered as a meaningful gestalt and not as a mere aggregate of “mental objects,” phenomenological psychiatry overcomes the reductionist view of mainstream psychiatry, in which symptoms are considered as independent object-like entities, which can be objectively measured independently from each other (3, 13–15).

The phenomenological method thus already introduces a fundamental systemic principle when it comes to explaining psychological symptoms: Every experience and symptom cannot be explained acontextually but must be always viewed with regard to the meanings that it derives from its eidetic contexts and structure of experience in its temporal and dynamic unfolding.

### Phenomenological psychiatry and the problem of individualism

Although –methodologically- phenomenological psychiatry stresses the importance of contextualizing single symptoms and experiences within a systemic whole, the focus has still been mainly limited to the individual person. There have been notable accounts, which have especially analyzed the relevance of intersubjectivity for the constitution of experience and have taken intersubjective factors into consideration in processes of static and genetic understanding. The dimension of intersubjective experience was in fact present in phenomenological descriptions of mental disorders right from the very start, e.g., in all authors from the so-called “Wengener Circle,” i.e., Binswanger, Minkowski, von Gebsattel and Straus (16). However, the intersubjective dimension was and still mostly is considered as a quality of the *individual‘s* experience and *their* being-in-the-world. Intersubjective factors hence have been mainly considered to be the byproducts of a primary disturbance of experience, which originates and resides within the individual^4^. Phenomenological eidetic explanation has thus mainly focused on the individual person rather than her social context^5^.

For example, the current predominant theory of schizophrenia in the field of phenomenological psychiatry conceives it as a disorder of the minimal self (22, 23). According to this account, schizophrenic symptoms are rooted in a disturbance of self-affection, i.e., someone's basic, pre-intentional and vital sense of self, i.e., the minimal self. The essence or structure of a mental disorder such as schizophrenia is therefore in this case viewed as residing *within* the individual, i.e., a loss of self affection or diminishment of the minimal self. As a consequence, this account tends to bracket social factors of schizophrenic experience–factors that would come to the fore by looking at the embeddedness of self-experience into the *world* via manifold forms of interaction. This contemporary phenomenological account of schizophrenia thereby seems to confirm the well-known and almost classical criticism expressed toward phenomenological psychiatry: it remains limited within a narrow individualistic perspective, thus the social aspects are secondary and not constitutive of lived experience (24–27)^6^.

But how come that in the meaningful whole of experience and its dynamic unfolding such intersubjective factors are so downplayed? Considering that the explanatory power of phenomenological psychiatry lies in contextualizing single symptoms within an holistic gestalt of experience this individualistic tendency appears surprising. For if single symptoms experienced by an individual are to be explained through this individual's whole of experience, why should phenomenology then as a next step not try to explain this individual structure through its broader context, i.e., the structure of its relational and social world? In other words: why should contextualizing eidetic explanations stop at the individual level?^7^

Another important consequence of downplaying intersubjective aspects in the analysis of experience becomes evident when considering explanations beyond the phenomenological domain, i.e., the determination of experience by processes outside consciousness in terms of explanatory psychology (2). According to Sass and Parnas (11) and Sass (32), in the case of schizophrenia, the experiential basic disturbance of the minimal self is seen – from a genetic explanatory perspective - as primary, since it concerns the most basic act of awareness, which is conceived as being the foundational level of the self (33). By conceiving the minimal self as primary and foundational for consciousness and experience, the authors conclude that the disruption of this level might “be a rather direct consequence of a neurally based cognitive dysfunction” (11, 32). They thus encourage empirical research looking for the neural correlates of self-disorders, as a future direction for phenomenologically informed research on the pathogenesis of schizophrenia.

It here seems that the physicalist approach of biological psychiatry that was rejected from the front door of phenomenology, returns from the back door after all. Although in Sass's and Parnas's (11) proposal, subjective experience is not reduced to a mere epiphenomenon of neurophysiology (as they recognize even the causal relevance of phenomenological processes), the “ultimate causal primacy” for the basic experiential abnormalities of the minimal self is indeed located in neurobiological abnormalities in the brain (11)^8^. It thus seems surprising how the two authors claim an explanatory power of context (in terms of phenomenological structures and processes) but then in fact stop at the individual level (and finally the individual brain) – instead of continuing by contextualizing this eidos in broader fields of explanations, i.e. *social* structures of being and interaction.

In contrast to these individualistic tendencies in phenomenological psychiatry, Wolfgang Blankenburg broadened the scope of eidetic explanation, i.e., he looked for intersubjective structures from which individual experience and symptoms may emerge (and to which they may react to). In the following and main section of this paper we will present this project.

## Part 2. Expanding phenomenological psychiatry toward the social: Wolfgang Blankenburg's approach

The work of Wolfgang Blankenburg and especially his research on schizophrenia is a common reference for many contemporary phenomenological psychiatrists. One of his key concepts on which current phenomenological authors have drawn (36–39) is that of a *loss of common sense* (or loss of natural self-evidence) as a typical modification of experience in schizophrenia (40, 41). With this concept, Blankenburg tried to capture a difficulty or inability to naturally engage in everyday social interactions and to pragmatically access the world. This is shown for instance in difficulties to spontaneously read between the lines of what others say and express, which has also been traditionally described as “schizophrenic autism” (38, 39).

Blankenburg developed this notion especially in the *The loss of natural self-evidence*, a single-case study about the experience–both inside and outside of clinical contexts–of the patient Anne Rau, a person with a hebephrenic schizophrenia (40). One of the main results of Blankenburg's phenomenological analysis is that the loss of common sense does not only play out at the level of intentional thinking but also at the intercorporeal dimension of embodied and intuitive interaction. Blankenburg's analysis here echoes classical phenomenological concepts such as Minkowski's “rationalisme morbide,” “affaiblissement pragmatique” (42) and Binswanger's “schizophrenic eccentricity” (40, 43). In the same vein as these authors, Blankenburg tries to give a detailed account of the structure or *eidos* of Rau's schizophrenic experience, which he classified in four experiential dimensions: world, time, ego/selfhood and intersubjectivity (40).

Although Blankenburg's eidetic analysis remains mainly on the level of *static understanding* (see above) he also tried to *genetically understand* how the structure of Rau's experience arose from her biographical background and familial socialization. When describing what Rau herself later called natural self-evidence, she indeed often refers to her biographical and familiar background, as for instance in this quote: “Only mommy can give this to me. Or it must be a family, who gives back this naturality” (40). For Blankenburg here “mommy” does not stand for Rau's own mother (with whom she describes a rather troubled relation), but more generally for the socializing function of family, i.e., the mediation between an individual person and her social surroundings. Blankenburg refers here to the emergence of a “basic trust” as the foundation of any interactional relation of an individual with her surroundings (40). His analysis leaves the question open as to how such a loss (or re-establishment) of trust and self-evidence toward the world might concretely occur.

Current phenomenological accounts of schizophrenia–as the ones we have presented above–integrate Blankenburg's intersubjective notion of loss of common-sense and reinterpret it as a consequence of a subjective disturbance of minimal self-experience. Thereby they decontextualise the subject from the practical-social life processes. In contrast, Blankenburg believed that an understanding of schizophrenia as loss of common sense must go beyond the individual structures of consciousness and recognize the intersubjective constitution of different worlds and socio-cultural contexts underpinning the individual experience of patients. In other words, Blankenburg claimed that the concept of common sense and of its loss in schizophrenia calls for an analysis not only of the self but even more of the social and cultural context, i.e., the different *lifeworlds* in which the constitution of everydayness is inhibited (12, 40, 44, 45). Especially the later works of Wolfgang Blankenburg, with their strong orientation toward the lifeworld of patients, are a notable example of how a phenomenological analysis may take into account the social dimension and its explanatory power in the case of schizophrenia and more generally for mental disorders.

The expansion of phenomenological psychopathology into a more social and–one might argue–systemic direction can be described in the work of Wolfgang Blankenburg at three different and yet related levels. At the conceptual level, he emphasized the importance of theories from phenomenological sociology and ethnopsychiatry, in order to understand the social constitution of experience and the cultural and sub-cultural specificity of interactional norms (and the loss of sense for them). At the empirical level, Blankenburg et al. (46), studied the evolution of schizophrenic experience within the familial context: he differentiated between different family milieus related to specific structures of meaning and explored possible therapeutic means for recovery. At the methodological level, Blankenburg argued for a dialectical perspective, inviting professionals to understand schizophrenic experience and symptoms as a result of the interaction with the surrounding world, thereby questioning professionals' individualizing and deficitary gaze. In what follows we outline and discuss these three perspectives of Blankenburg's work.

### The conceptual expansion: Focusing on the social structures of the lifeworld

We have previously introduced eidetic phenomenological psychiatry as a method of contextualizing and thus explaining single symptoms within a structural whole of experience. We then critically discussed the application of this method in the history of phenomenological psychiatry, which has been limited to the individual level. However, Husserl in his later works on the constitution of lifeworlds [(47), cf. (48)] shows how trans-individual and even transcultural structures of intersubjectivity are accessible to our individual experience. Phenomenological sociology as a discipline founded by Alfred Schütz^9^ focuses exactly on such overarching structures of social interaction in which our subjectivity is embedded. The basic idea here is that these structures–in terms of sedimented knowledge and typifications of the world and implicit rules of interaction–are both produced and *re*produced by our social and intersubjective experience, that they serve as context that both forms this experience and is formed by this experience–finally constituting our experience of a commonly shared normality and “paramount reality” (50)^10^.

Drawing on the tradition of phenomenological sociology, Blankenburg conceptually extended the field of phenomenological psychopathology so as to enable a phenomenology of the social, beyond the individual (45, 52, 53). Indeed, through the lenses of phenomenological sociology he explored how the structure of individual experience might be affected by the structure of broader social contexts. Importantly, for Blankenburg these social contexts and structures are not considered as being outside of consciousness but, on the contrary, as being *phenomenologically* accessible.

The central question for him was more specifically how the experience of reality of persons with mental disorders might be constructed as a deviation from the above mentioned shared common sense-normality in mutual constitutive processes between them, the environment and also the psychiatrist (44, 45, 53).

Adopting the perspective of phenomenological sociology thus lead him away from the individual to the constitution of more general and social structures of interaction, or as he puts it:

“This line of questioning leads from the reality experienced and shaped by the patient back to subjective and intersubjective processes of reality that are to be traced for the individual as well as for his or her family and (historically) for entire societies” (12).

This conceptual expansion toward a more socially oriented and systemic approach in Blankenburg's work was not only based on phenomenological sociology but, importantly, also on an ethnopsychiatric perspective, i.e., the field of transcultural psychiatry [(54), cf. (55)]. In his essay *Ethnopsychiatry in the homecountry* [Ethnopsychiatrie im Inland] (56) he shows how the natural self-evidence and common sense varies in different subcultures and communities. He thus argues that a diagnosis of a mental disorder should be paralleled by an understanding of such a community, e.g., through an ethnographic investigation.

Blankenburg for instance reports the case of a farmer's son, brought to a psychiatric hospital, who believed he was capable–by divine intervention–of turning water into gasoline and to chase the “evil one” out of the farm's stable (56). Although a psychiatrist at first glance might immediately take the patient's experience as entirely delusional, Blankenburg noticed that the father, too, believed in “the evil one” and he himself had already tried to catch it in his stable. Instead of also being delusional, this belief, as many others reported by the patient's father, was part of the village community's shared and common superstitions. Blankenburg thus here differentiates between the conviction to chase the “evil one” out of the barn and the conviction to be able to turn water into gasoline: the first *is socially accessible and shared*, the latter is *not*–rendering the first a mere expression of a socially shared normality and the latter a sign of a pathological loss of this very shared sense of normality. Blankenburg's patient consequently was diagnosed with schizophrenia, received medical and psychotherapeutic treatment and was then reintegrated into his community where he continued working as a farmer (56). However, after the patient committed suicide, Blankenburg hypothesized that despite the treatment and the attempts at reintegration, he still had lost connection to his community.

The emphasis on the importance of social factors for the constitution of and recovery from schizophrenia becomes immediately evident in this example. Another important point is that the distinction between what is normal and what is pathological is not universal, but always relative to the concrete norms and values of a social group and, moreover, to a person's capacity to communicate with these norms. Persons therefore have to grow into these specific norms in order to develop such a sense (i.e., a common sense). The central social institution mediating this process of growing into a social community in Modern Western culture is the family (57). Consequently, the loss of common sense for meaning and interaction can in fact not sufficiently be studied by focusing on the individual experience of the patient – the phenomenological perspective must be extended to the structures of families and subcultures underpinning a person's connection and integration into social interaction. This is the point where Blankenburg's empirical expansion of the phenomenological approach toward the study of family milieus and subcultures sets in, which we will present in the following section.

### Empirical expansion: Studying families of adolescents with schizophrenia

Blankenburg's empirical research on the social constituents of schizophrenic experience is probably one of the most neglected and unnoticed parts of his work, especially at the international level. One reason might be that Blankenburg himself only rarely referred to this research in his publications. Yet, this is surprising since, as we have argued so far, such empirical research appears as a logical consequence of Blankenburg's conceptual reflections.

Since his seminal case study on Anne Rau, Blankenburg's research on schizophrenia focused on adolescents and their families (40, 58). Blankenburg diagnosed Anne Rau as suffering from hebephrenic schizophrenia which (as derived from the Goddess of youth “Hebe” in ancient Greek religion) typically appears in adolescents and young adults (58). Blankenburg considered adolescence “a decisive stage of ego-development” where a person has to position herself toward her social milieu in terms of a ‘psycho-social self-definition”' [(58); see also already in (40)]. For him it was thus important to study the *familial context* of those critical cases, where the attempts at such a self-definition coincided with the onset of schizophrenic symptoms. This interest in the familial context also seems to be fuelled by Blankenburg's clinical experience, as he reports: “It is always a deeply moving event when we are able to break through the seemingly extraordinarily ‘endogenous' behavior of an adolescent schizophrenic and suddenly discover the bitter seriousness of a life-story problem that could hardly have been guessed at before. [...] In the place of the facetiousness and silly-lappy appearance, a previously completely concealed deep despair suddenly emerges” (58).

One might indeed argue that Blankenburg drew his motivation for understanding patients' social and familial situation not primarily from theoretical reflections but most of all from his clinical experience and encounters with patients. It is here that he saw necessity for broadening the field of explanation to the social sphere, which thus lead him to engage both conceptually and empirically with the field of sociology.

In 1980 Blankenburg hired the trained sociologist Bruno Hildenbrand, who was involved both in clinical and scientific work. In the same year they received funding from the *German Research Foundation* (*Deutsche Forschungsgemeinschaft, DFG*) for a research project entitled “Family situations and orientation of schizophrenics toward the everyday-world” [“Familiensituation und Alltagsweltliche Orientierung Schizophrener,” (46)]. The core idea of the project already becomes evident in the title, namely that family context and orientation toward the everyday-world (i.e., the sense of shared normality or common sense) are connected. As Blankenburg explains:

“The question of disturbances in the orientation toward the everyday-world raises the question of the patient's practice, i.e., how does the patient–in interaction with his closest caregivers, with his family or at work, etc.–construct the world? How is the world constituted for him? How does he constitute the framework in which he encounters the world and himself?” (44)

In order to address this complex question, Blankenburg, Hildenbrand and their colleagues analyzed meaning structures of different families of patients with schizophrenia. They used narrative interviews with family-members about the family-history as empirical methods. This was combined with sociodemographic information about three family generations. Moreover, Blankenburg and his colleagues used participatory observation at home and in the lifeworld of these families, thereby aiming at the description of implicit and explicit rules of interaction and sense-making within the familial and broader social milieu of the participants (46). The researchers also accompanied the participants when moving through the different domains of their lifeworld and simultaneously interviewed them about these domains–a method that today is known as *go alongs* (59). As methodological framework for their empirical investigation, Blankenburg and his colleagues chose a *grounded theory* approach (60), which allowed them to integrate the aforementioned empirical methods. Within this methodological framework, families were recruited for the study in a reiterative process of *Theoretical Sampling* (60) until empirical saturation of the developed concepts was reached^11^.

A first aim of the study was to describe typical meaning structures in the families. Yet the focus of the research project, influenced by phenomenological sociology, went beyond the interaction of the patient with his family. The family situation was studied both in its biographical and its specific societal situation, on the basis of which the specific meaning structure of the family was reconstructed (46).

Generally, the results of this study showed that adolescents with schizophrenia experienced a failed process of emancipation from the family and a failed integration into society with its more general and anonymous structures of meaning and interaction (57). Internal-external mediation was identified as a central structural problem in families of adolescents with schizophrenia, i.e., a mediation between the family's private life and space and the external and anonymous social world, serving as the basis for a person's emancipation from her family and allowing or inhibiting her new positioning in the external social world [(62); see also (63)]. Blankenburg and his colleagues observed three different types of structures of meaning or family-milieus characterized by a failed mediation between the inside and the outside of the family system. These structures of meaning were present in the family at the onset of schizophrenia in adolescence (62)^12^.

*The outwardly demarcated and inwardly centered family milieu* *(**62**)*

This appeared as the type of family most likely to be found in the case of persons with schizophrenia. It is characterized by the fact that the family-specific construction of reality is at best fragmentarily conveyed to the social environment, the family members cannot adequately relate to societal conditions and to their changes and they are therefore societally isolated.

*The outwardly oriented and inwardly disclosed family milieu* *(**62**)*

This type of family was mainly found among small self-employed people in the tertiary sector, e.g., hotels or restaurants. Family life is reduced to formal relationships with highly structured interaction contexts. Relationships within the family consist of formal working relationships. Milieus directly related to the family in the sense of a non-business-related network of the family, which could compensate for the lack of milieu-like forms of interaction, are largely absent. Children in these families did not succeed in settling down permanently outside the family, although they constantly made attempts to do so.

*The family milieu with a contradictory inner-outer orientation* *(**62**)*

In this type of family, patterns of orientation and action can be found that are directed toward an increased orientation toward the outside world. In the foreground is the striving for social ascent, which the parents were not able to realize for themselves, or only partially, and which they delegate to their children. This increased external orientation corresponds to a separation from the immediate surroundings of the family, i.e., the village, to whose traditional structures the family is also oriented. The latter is inevitable because the world outside the village, toward which the family–striving for ascent–is oriented, is nevertheless alien and opaque. Two opposing patterns of inner/outer orientation are thus represented simultaneously, without the family being able to decide on one or to develop a practical pattern of orientation and action from both. The children are bound to the traditional patterns of orientation and action in the long term and fail on the path of ascent delegated to them.

Blankenburg and his colleagues explored these family milieus in relation to the evolution of the schizophrenic disorder and drew reflections for possible therapeutic consequences. In Germany, patients with schizophrenia after being hospitalized were often referred to other institutions such as residential family homes (Familienheime), therapeutic communities or other residential homes. Based on the idea that schizophrenia from a socio-dynamic point of view represents a form of failed emancipation from private family-structures into the social and public world, Blankenburg and colleagues thus empirically investigated how these therapeutic institutions might enable or support this delicate transitory process (46, 64). Their basic idea was to consider residential homes as a “therapeutic instrument with specific possible risks and chances” for rehabilitation (64). In the second part of their empirical research project they focused on three types of institutions: first, a transitional facility, which had the structural features of a family home; second, a therapeutic living community founded by a psychologist; and third, a transitional residence centered around the client-therapist relationship and at the same time structuring everyday life through an “explicit, bureaucratically determined therapy programme” (62, 65).

Emancipation means crossing a boundary. To emancipate oneself from what is given in order to construct one‘s own adult identity and develop a sense for the natural self-evidence can first require the establishment of routined actions and orientations. Among the three institutions under study, Hildenbrand identified the family home as the closest to what he then called a “enacted/staged family” (62). Family homes are small institutions in which up to about ten persons with mental disorders are cared for in the house of a family. They are usually run by the housewife, who is trained as a nurse. It is thus a kind of “artificial family”: it is characterized both by private elements of daily being together and trusting each other (in the sense of classical family interaction), and by public elements, since the people employed in the family home perform this function as a professional role with corresponding specifications in a public health facility, alongside their private lives, and do so only for a limited time. One could therefore speak of a paradox of a “temporary family” (62), which enables the residents or patients to make a transition, forcing “permanent negotiation processes between the manager, the residents and the manager's relatives” in the protected area of the family, which offers “opportunities for manifold boundary negotiations” (62). Family homes therefore appeared as especially helpful for persons with schizophrenia to achieve a transition from the family world to the external social world. A problem however was, that this process of transition wasn't therapeutically supervised or integrated, i.e., that there were no professional therapists present at this process in order to circumvent potential pitfalls or failures, as Blankenburg and his colleagues concluded (64). They considered as a consequence of this lack of therapeutic reflection the fact that many residents often and again failed to emancipate themselves from these structures and to orient themselves toward the external world (e.g., live in their own apartment, have a job outside of the institution).

### Methodological expansion: A dialectical perspective

Blankenburg's conceptual and empirical expansions of the phenomenological approach were paralleled by a methodological transformation. Blankenburg's introduced and integrated a dialectical perspective which he tried to integrate into psychopathology and therapy–an attempt that is already perceptible in *the loss of natural self-evidence* (40), but more thoroughly explicated in several subsequent studies [(66–68); see also (69)]. Blankenburg's central motive is that in order to understand the nature of a person's suffering it is necessary to take mental disorders not merely as a deficit but as a possible meaningful reaction to and at the same time as negation of a certain norm of health (68): more specifically, as the individual's reaction to challenges in the process of becoming autonomous. Blankenburg makes this claim about mental disorders in general but applies it especially to schizophrenia [see already in (40)]. Thereby Blankenburg's aim is to highlight the positive and creative aspects of mental disorders and to shift the clinician's perspective from a mere and passive suffering to one in that recognizes that a person always makes something out of what she is made into, i.e., that there is always also freedom in mental disorder [cf. (70, 71)]. Blankenburg thus calls for the clinician to change her own perspective and to get rid of a rigid and deficitary view of patients. He calls this negative orientation “an orientation toward the minus,” which is usually most common for psychiatry's understanding of mental “illness” (40). In other words, Blankenburg invites psychiatrists to think of mental disorders not as something that can be diagnosed independently of a specific form and norm of mental health: on the contrary, mental disorders are a dialectical and creative reaction to mental health^13^.

Another example for a dialectical approach is Blankenburg's concept of the dynamic relation between biography and illness (67, 68, 73). First, he invites professionals to refrain from only detecting deficits and disorders and to view patients' medical history within the context of their biography. Instead of reducing the medical history to a sequence of symptoms, diagnoses and treatments, it should be viewed as an attempt to cope with biographical challenges and thus in constant exchange with personal biography and its meaning. In such a biographical and meaningful context, illness can then be conceived as a dialectical anti-thesis in terms of a *crisis* and *hiatus* in the continuity of someone's life story, calling out for a decision and for projecting a new future. The way in which this decision is made (or avoided) will in return have repercussions on the course of the crisis. Mental disorders and biography thus are to be considered in a reciprocal and dynamic process.

Such a dialectical perspective also has therapeutic implications. Indeed, looking at a mental disorder from the perspective of a possible new future (instead of from the perspective of an unchangeable past) could help patients to actually overcome critical phases. Blankenburg (73) called this attempt the “future perfect-perspective.” An example would be to ask patients what use they believed their mental disorder will have had 1 day in the process of becoming autonomous (73). This question actually has two effects, the first being that it can help patients to stop looking at their crisis only from a deficitary point of view in terms of an illness, but instead as something endowed with meaning and potential for further autonomy, since it is embedded in the meaningful context and unfolding of their biography and autonomy. The second effect is that this question can haul them out of their current incapacity to envision an alternative future of their biography (or even a future in general, as is often the case in mental crises) to which the current crisis could also contribute.

In Blankenburg's dialectical approach it also became clear that his explanatory claims follow a teleological rather than etiological logic. Schizophrenic symptoms and experience are not assumed to stem from certain *causes*, yet their emergence is justified within a framework of *reasons and responses*, i.e., to what context can the symptom or experience be considered a meaningful and adaptive reaction (68). Blankenburg acknowledged and stressed the multi-factorial and complex processes that might explain the emergence of the disorder, yet with his approach he particularly recognized the relevance of – and explicitly focused on - the contextual social structures (46, 68, 69, 74). This is in contrast to several phenomenological authors, which – when looking outside the domain of phenomenology – have rather been focused on and encouraged research in the neurobiological field (11, 75–77).

Last but not least, Blankenburg generally favored a qualitative and idiographic approach, which allows for an in-depth case-to-case analysis and understanding of individual lifeworlds. Refraining from etiological claims, he believed that such an understanding could sensitize clinicians to be sensible to e.g., familial structures of sense-making and could stimulate further research (46, 74).

## Part 3: Toward a systemic phenomenological psychiatry

Blankenburg's expansions of phenomenological psychopathology thus put forward a relational and contextual perspective, in which individual symptoms and disturbed experiences are viewed in constant exchange with the social background, reciprocally conditioning one another. Mental disorders aren't thus located within the individual, but necessarily arise from the interplay between individual, social and cultural factors. This relational view in Blankenburg's work is coherent with a systemic understanding of human experience, identity construction and mental disorders. In this last section we thus emphasize the systemic elements present in Blankenburg's work and the links to the field of systemic therapy, and at the same time, also briefly discuss some points of divergence. Finally, we also draw implications for the topic of explanation, suggesting that Blankenburg expanded phenomenological explanatory modes into a systemic direction.

The links and interfaces between Blankenburg's work and current systemic approaches begin at the conceptual level. By expanding his theoretical background to phenomenological sociology Blankenburg drew on seminal works like Berger and Luckmann's *The social construction of reality* (78), which also constitute an important theoretical basis for systemic therapy, especially in the socio-phenomenological tradition, focussing on the social construction of reality rather than looking like radical constructivists at the individual construction of individuality (79). The idea that the subjective experience of a person is always intersubjectively and biographical constituted thus seems to be a first important theoretical common ground.

Blankenburg and his colleagues' research on families with adolescents with schizophrenia is a special case in point: The subjective experience of schizophrenia is tightly related to specific intersubjective family structures and sense-making. Therefore, according to Blankenburg, the phenomenology of schizophrenia is not confined within the individual: On the contrary, it is intersubjectively constituted and embedded (46). A phenomenological investigation thus needs to integrate different perspectives, i.e., of the patient, of the relevant others (e.g., family members), of a cultural background and last but not least, also of the researcher.

Early systemic authors such as Jackson, Haley, Watzlawick and Bateson were highly influenced by Harry Stack Sullivan's *Interpersonal Theory of Psychiatry* (80), who devoted years of clinical and research work to help people with psychotic illnesses, especially with schizophrenia. He was the first psychiatrist to introduce the groundbreaking idea that all psychological disorders have an interpersonal origin and can be understood only with reference to the patient's relational and social context. Consistently with current tenets on psychosis, Sullivan theorized that psychotic breakdown was related to severe interpersonal interference with the person's “self-system,” later developed by Laing (81) into *The Divided Self* . The idea of schizophrenia as an extreme defensive identity reaction against family disconfirmatory communication patterns is the core tenet of the early different systemic contributions on the topic, which links the various constructs introduced by each author (Bateson's double bind; Wynne's pseudomutuality; Laing's disconfirmation and mystification …).

More than with these communication-oriented models of psychotic family interactions and those further developed by the Palo Alto group (82–84) and by the Milan Approach (85, 86), Blankenburg's explanatory hypothesis relates schizophrenia to specific family structures of meaning that hinder the processes of emancipation. Thus, it strongly resonates with the systemic tradition of multigenerational family therapy, e.g., in the work of Stierlin (63), Bowen (87), Boszormenyi-Nagy and Spark (88).

Similarly, in their family studies, Blankenburg et al. (46) propose that in the case of schizophrenia certain family structures of meaning may hinder the patient's emancipation from them. As a consequence, also the transition into more socially shared and public structures is hindered, which might in turn explain the diminished sense for a shared normality, i.e., common sense. Schizophrenia can thus be understood within the context of a problem or a struggle residing at the broader social level of the family and of her relation to the outer society. In this sense, Blankenburg even suggested to conceive schizophrenia as an attempted solution to a problem in social structures, more specifically to the problem and challenge of emancipation from a specific family milieu (58, 68, 69)^14^.

Indeed, Blankenburg and colleagues refer to this literature as part of the theoretical background for their family studies. One of the central concepts in Stierlin's work (63) is *related individuation*, which refers to the possibility of emancipating oneself without breaking up contact, i.e., a middle ground between what he calls the two transactional modes of binding (with a consequent negation of emancipation) and expelling (with a consequent negation of relation). Related individuation thus means for Stierlin a successful process of emancipation from the original family. If this is hindered, psychological symptoms can arise, which he then also more thoroughly investigates and differentiates in terms of specific familial and multigenerational factors. Drawing on these concepts, some of the current systemic explanatory accounts of schizophrenia similarly conceive of schizophrenia as arising from a problem of emancipation from the original family (89, 90)^15^.

Current systemic contributions such as Linares' (91) have abandoned the quest for a specific family structure that can be associated to the onset of psychotic disorders; the family interactional and communicational patterns in the here and now are still considered to be more emblematic of these families' functioning; they are still a source for inspiration and the main therapeutic resource for systemic interventions in these psychopathologies, although very different from early ones, such as counter-paradoxes and therapeutic double binds. Most systemic theories of psychotic disorders still revolve around the triangulatory and hidden process of disconfirmation and disqualification, which, by denying acknowledgment of the patient, undermines their psychological existence and hence their very identity. This is especially dramatic when the family life cycle approaches the stage of the young person's “emancipation” or better, differentiation, and thus the definition of their own identity: an impossible step for the patient's positioning within these family contexts dominated by disconfirmatory patterns.

Drawing on Batesonian intuitions on relational context and meaning, as well as on socio-constructionist developments of the original concept of double bind [i.e., strange reflexive loops, (92)] and Positioning Theory, Ugazio's (79) systemic theory of family semantic polarities fills the gap, linking together each family meaning making with individual positioning (identity) within these conversational contexts. She is able to explain how one person can become ill and not the others: the onset of psychopathology is triggered by the unique and reciprocal positioning that the individual patient and their family members take within the critical meaning in their broader socio-cultural context. By co-positioning along the shared plot of family semantic polarities, each family member binds their identity to the identities of other members occupying different positions: it anchors their interdependent Selves or identities from the very beginning. This intersubjective acknowledgment and recognition is not granted to the future psychotic member.

Thus, current systemic therapeutic approaches to psychosis (91) will systematically promote acknowledgment through a reconfirmation process: although this is carried out differently across different models, it always entails an extended group conversation (family session; Open Dialogue; Soteria house) that finally allows the intersubjective reconfirmation and validation of patient's identity, which was previously denied to them.

Another common ground between Blankenburg's work and systemic approaches is to be found at what we called the methodological level, i.e., the dialectical perspective. By taking a dialectical perspective Blankenburg describes mental disorders in general, and schizophrenia in particular (e.g., in his family studies), as meaningful reactions to certain difficult social circumstances (68). It is thus never about a pathological experience *per se*, but rather about the circular relation between such experience and its context.

Similarly, from a systemic perspective, the key to explaining a symptom is, in the first place, to broaden the inferential field and put it into context (93, 94). We have mentioned in the introduction how explanation through contextualization actually characterizes the very phenomenological method (95). Yet, we argued that Blankenburg, by extending the contextual field beyond the individual (and the dyad), has brought phenomenology even a step closer to this systemic perspective.

A dialectical approach on symptoms as meaningful reactions to difficult situations does not only emphasize their embedded and relational character, but also resonates with another core principle of systemic therapies, namely the idea of symptoms as solution attempts (93, 94). A basic tenet of systemic therapy is that symptoms are constructed as “unconscious” creative - and adaptive - attempts to tackle difficult or even paradoxical relational situations: it thus positively reframes what is usually viewed as a deficit (96, 97). According to this perspective, a patient is thus not reduced to a cluster of symptoms: rather she is viewed as a competent and sensitive individual, thereby automatically shifting the focus from the deficits to the resources and agency of the person. This leads to a depathologizing, empowering and respectful clinical stance, which, as we have outlined above, is notably present also in Blankenburg's work. We have also seen how one consequence of such perspective is a particular orientation toward the future. The imagination and (thereby) actualization of possible future solution scenarios, which takes in Blankenburg's approach the form of a “future perfect-perspective,” has the effect of expanding possibilities, activating resources, strengthening motivation for change and finally helping her to unfold her autonomy. Interestingly enough, this is also a typical method of systemic therapy (98).

Blankenburg's dialectical perspective thus entails several aspects, which are in common with a systemic approach. From these aspects a particular kind of explanatory modality might emerge, which is not only focussed on the past, but also oriented toward the present or future: indeed, by understanding and explaining mental disorders as meaningful reactions and even solution attempts to difficult circumstances the focus is not on the “why,” but on the “what for.” Thus, as also outlined above, in his phenomenological account, Blankenburg did not only extend the field of explanatory inference to the social structures but – one might argue – he also pointed toward a teleological explanatory mode, which focuses on the motivation and reasons instead of the causes (etiology) (74). Once more, Blankenburg's work thus aligns with recent systemic therapy contributions focused on meaning making (91–93).

Yet, despite the important similarities, a notable difference between Blankenburg's and systemic explanations is worth mentioning. Indeed, although Blankenburg recognized the meaningfulness of symptoms within their social context, he still sees schizophrenia as a *loss* of common sense, i.e., as a pathological loss of the shared sense of normality (see above, e.g. p. 5). Going back to the farmer's son example in section Phenomenological Psychiatry and the Problem of Individualism, from a systemic perspective one would not only assume that psychotic responses make sense (are coherent and “logical”) within their context's meaning making and communicational patterns–as Blankenburg also does–but also that the perceived loss of meaning is only apparent or temporary. A systemic approach would thus maintain that the farmer's son in fact still has a “sense of shared normality” but that he needs to communicate in different ways that prima facie appear incomprehensible. The non-accessibility of communication is thus not to be conceived as a “loss” of something but–also here–as a creative solution: e.g., it's a paradoxical and metaphoric communication if metacommunication is not possible (see original double bind concept and its revisitations such as strange reflexive loop). In other words, instead of being a proof of loss of sense for shared normality, schizophrenic symptoms quite to the contrary testify to a strong sensitivity for this normality and its paradoxes.

Given the many points of contact and similarities with a systemic approach one might expect a development of Blankenburg's research and clinical work toward a systemic direction. Interestingly, however, Blankenburg did not follow this route. Although taking part in social psychiatric discussions in the 1990's, his remarks remained by and large restricted to the relation between patient and psychiatrist, with little reference to the aforementioned family studies. Despite his strong sense for the importance of the connection between phenomenological and social and systemic approaches in psychiatry–which is also evident in the extensive literature on the topic he cites–in the last years of his research Blankenburg was more interested in questions of emotivity (99) and temporality (100) and further methodological questions in psychiatry (101).

It is Bruno Hildenbrand who expanded Blankenburg's approach in a systemic direction by a more thorough investigation of social conditions of mental disorders and their therapeutic implications (102–104). By embedding schizophrenic experience into a family milieu that hinders emancipation, Hildebrand especially focuses on institutions that help patients making up for this transitory process and learning to navigate and orient themselves between different structures of interaction (e.g., private and public spaces and interactions). He aims at better understanding how to develop such structures and evaluate their functioning (103). This clinical research resonates with current social psychiatric and systemic approaches to the treatment of schizophrenia like the Soteria Houses, which also aim at creating a safe family-like space for young people experiencing psychosis (105). More broadly Blankenburg's, and subsequently Hildenbrand's, research point to the need of explanatory models and treatment approaches that include (and thus also use the resources of) patients' social context, as it is for instance done in the social network approach of Open Dialogue (106), in which not the individual but her network is seen as the key to understanding and finding new solutions to the patient's problem.

Ultimately, one should also note that not only phenomenological psychiatry has much to gain from systemic approaches but that these approaches, too, may profit from phenomenology. The phenomenological perspective indeed provides an account of the what-it-is-likeness of experience from a first and second person perspective (91), which have been for long time missing in the systemic approach. Even if in more recent constructivist and social constructionist systemic approaches the experience of the person is taken in consideration, most of these approaches are concerned with the narrative level. Phenomenology thus may offer a deeper and more nuanced account that includes the more basic experiential structures of subjectivity and of its social embeddedness.

In this paper we have focussed on the field of phenomenological psychopathology with the question of what types of explanatory modes are put forward in it. First, we showed that phenomenology indeed entails explanatory elements and that those follow a–one might argue–systemic principle of contextualization. Yet, when looking at the inference field of its explanatory accounts, phenomenology has remained mostly individualistic. We thus presented Blankenburg's work as overcoming this impasse and extending phenomenological explanation to broader social and cultural structures of experience. In this sense, we believe that Blankenburg's work is of extreme relevance for the current developments in phenomenological psychopathology and that it may also point out opportunities for further exchange between current phenomenological and systemic thinking in psychiatry.

## Author contributions

ST and LG wrote the manuscript, which was revised and approved by MK and LF. All authors contributed to the article and approved the submitted version.

## Funding

This publication was funded by the MHB publication fund supported by DFG.

## Conflict of interest

The authors declare that the research was conducted in the absence of any commercial or financial relationships that could be construed as a potential conflict of interest.

## Publisher's note

All claims expressed in this article are solely those of the authors and do not necessarily represent those of their affiliated organizations, or those of the publisher, the editors and the reviewers. Any product that may be evaluated in this article, or claim that may be made by its manufacturer, is not guaranteed or endorsed by the publisher.

## Footnotes

^1^It is important to mention that Jaspers' use of phenomenology in psychopathology was restricted to a description of single psychic states without trying to determine broader eidetic structures of experience and their evolution over time – as phenomenological psychopathologists starting with Binswanger, Straus, von Gebsattel and Minkowski did from the 1920's on (5, 6). Jaspers later showed himself sceptical of this attempt (7).

^2^When looking at a painting, for instance, we never see single colours or lines but landscapes, faces or objects. We wouldn‘t however say that theses structures are *outside* the painting. They are in fact the painting itself in terms of an holistic form of manifold colours and lines, which wouldn‘t ever become a painting as a whole if looked at separately. Another example are emotional expressions: When somebody smiles at us we never only see moving lips and cheeks but the immediate expression of friendliness. Again we wouldn‘t claim this friendliness is external to the facial movements. Friendliness is the internal structure of those movements. It is what holds them together.

^3^See also Romano's concise analysis on holism of experience in phenomenology (9).

^4^This becomes evident even in concepts opposing an individualistic stance, such as Binswanger's concept of being-beyond-the-world, which he developed in order to challenge Heidegger's focus on selfhood (17): In his Ellen West's case study, Binswanger (18) speaks of a lack of this being-beyond-the-world seemingly characterizing West's *Dasein* from the very start. For Binswanger, this lack is not constituted by West's social context, but is constituting this context instead. In his analysis, he thus neglects to give an extensive account of it.

^5^This focus of phenomenological psychiatry is, however, not a methodological one-way road preordained by phenomenological philosophy. Besides the branch of phenomenological sociology (see below, 2.2), especially the developments of phenomenology in France give proof of phenomenology's sociological potential. Here, phenomenologists such as Merleau-Ponty and especially Sartre intensely grappled with questions of social environment and collectives, ultimately linking phenomenology with Marxism [cf. (19)]. This is probably the reason why critical psychiatrists, such as Franco Basaglia in Italy or Klaus Dörner in Germany, eventually felt much closer to Sartre than to the phenomenological psychiatric canon (20, 21).

^6^Here one could also argue that the intersubjective dimension is very present in contemporary phenomenological analyses of schizophrenic experience, e.g. in the *Examination of Anomalous Self-Experience* [EASE, (23), see for instance Item 2.12] and even more so in the recent *Examination of Anomalous World Experience* [EAWE, (28)]. But again (see footnote 4) EASE and EAWE describe the *indivual's* self- and world-experience and not the world that this experience arises from (e.g. her family-context, social milieu, class, gender etc.). In other words, it is the individual that is focused on, interviewed and scored – not her family or her work place etc.

^7^One could say that Foucault's quarrel with phenomenological psychopathology was all about this problem. When he then left phenomenological psychiatry behind (24, 29) he in fact kept the concept of eidos and eidetic explanation well in mind and applied it to socio-historical phenomena (thus speaking of *historical apriori*) (30, 31).

^8^Interestingly, Sass's and Parnas's approach is in close to Huber's classical concept of “basic disorders close to substrate” [substratnahe Basisstörungen] (34): based on the phenomenological method, Huber and his colleagues described symptoms of chronic forms of schizophrenia and claimed that these basic symptoms were at the core of schizophrenic experience (35). They then believed that these symptoms were the closest that the analysis could get to finding neurological correlates–i.e., that it was finally the brain producing these symptoms.

^9^For a differentiation between phenomenological sociology and phenomenology see (49).

^10^In the same vein and based on ethnographic observations Thoma (51) has tried to show how the rules and orders of interaction of different social spaces (such as private or public space) are inscribed into our habitual experience and form the sense of our reality.

^11^At the time of the study (1983), *grounded theory*, which today has become a standard approach for qualitative research in Germany, was rather unknown to German researchers. Its implementation in the study was essentially promoted by Hildenbrand, who was a friend of one its founders, Anselm Strauss [cf. (61)].

^12^Since the final report of the DFG-project has never been officially published we will in the following cite Hildenbrand's official habilitation treatise essentially summarizing the project's results.

^13^It seems that Blankenburg's critique of psychiatric categories puts him in close company to antipsychiatric theories. Indeed, Blankenburg defends antipsychiatry against undifferentiated and vulgar refusal by mainstream-psychiatry, appreciating her dialectical critique of psychiatry, for instance by considering schizophrenia as a “healthy reaction to a pathological structure of society” (68). But Blankenburg is also sceptical of a perspective that merely changes the categories from top to bottom, *reducing* mental illness to mental health and mental health to mental illness, as traditional antipsychiatry sometimes does [(58), cf. (72)]. For Blankenburg, *a dialectical approach essentially is an open question*: It means to widen the clinical perspective towards other perspectives and to a wider context that might determine individual symptoms and phenomena without ever reducing one side to the other.

^14^Accordingly, Blankenburg explains: “The ‘strength' and ‘weakness' of an I must be seen and put into perspective with regard to the integration tasks that are set for a person. (...) Over-adaptation in these patients would then not so much be the direct expression of an absolute primary I-weakness, but rather the expression of a conscious or unconscious capitulation of trust in one's own power to shape oneself in the face of a task that is overwhelming from the outset, and thus the expression of an I that is only relatively too weak” (58).

^15^It is important to notice that by taking into account the broader social dynamics one should avoid the risk of pathologizing the family. Systemic thinking is indeed opposite to the idea of linear causality, i.e. that symptoms may causally be attributed to a deficit either in the person or outside the person (in the family). By now briefly discussing the Blankenburg's methodological expansion, we will show how this nonreductionist and depathologizing view is also to be found in his work.
